# Urinary Tract Infection in Patients with Urolithiasis: A Large Retrospective Observational Study of Clinical Features and Microbiological Spectrum

**DOI:** 10.3390/pathogens15010098

**Published:** 2026-01-16

**Authors:** Mehmet Erinmez, Mehmet Ozturk

**Affiliations:** 1 Department of Medical Microbiology, Faculty of Medicine, Gaziantep University, Gaziantep 27310, Türkiye; 2 Department of Urology, Faculty of Medicine, Gaziantep University, Gaziantep 27310, Türkiye; mehmetozturk000@gmail.com

**Keywords:** urolithiasis, urinary tract infection, urine culture, urine pH, ureteral stent, hydronephrosis

## Abstract

Urinary tract infections (UTIs) and urolithiasis exhibit a complex bidirectional relationship in which microbial colonization and urinary obstruction may mutually reinforce each other. This retrospective observational study evaluated clinical and microbiological factors associated with UTI in patients with urolithiasis using a large institutional dataset. A total of 23,241 urine cultures obtained from 12,708 unique patients were analyzed, comparing individuals with and without urolithiasis. In stone-forming patients, demographic variables, urine pH, hydronephrosis, ureteral double J stent presence and indwelling duration, urinary anomalies, and stone characteristics were assessed. Logistic regression identified independent associations, and ROC analysis defined optimal risk thresholds. UTI were more frequent in the stone group (34.5%) compared with non-stone forming patients (28.9%, *p* < 0.001). *Escherichia coli* was the most common uropathogen overall, whereas *Klebsiella pneumoniae*, *Enterococcus faecalis*, and *Pseudomonas aeruginosa* were significantly enriched in patients with stones. Elevated urine pH (OR: 6.37; CI: 2.67–15.19; *p* = 0.001) and hydronephrosis (OR: 9.14; CI: 3.74–22.35; *p* = 0.001) were independently associated with UTI. A stent dwell time above 29.5 days was associated with infection with 85% sensitivity and 54% specificity (AUC: 0.70; CI: 0.68–0.73), and urine pH 6.2 or higher was associated with infection with 86% sensitivity and 67% specificity (AUC: 0.77; CI: 0.75–0.80). These findings underscore that urine alkalinity, obstruction, and prolonged stenting are key factors associated with infection risk, supporting the need for careful stent management and timely microbiological evaluation in patients with urolithiasis.

## 1. Introduction

Urolithiasis, also referred to as urinary stone disease (USD), ranks among the most prevalent disorders of the urinary system [1]. A wide range of factors can contribute to the development of urinary stones, including elevated levels of calcium, urate, and oxalate in the urine; decreased excretion of citrate; changes in the pH and volume of the urine; anatomical anomalies; inadequate nutrition; and a warm workplace with accompanying dehydration [2]. Traditionally, the only well-established link between bacteria and urinary stones has been the association of urease-producing bacteria with urinary tract infections (UTI) and the formation of struvite (magnesium-ammonium-phosphate) stones [3]. However, since struvite stones account for only 4% of all urinary stones [4], this suggests that a significant aspect of the bacterial involvement in stone formation may have been overlooked. Recent evidence indicates a more extensive association between bacterial presence and urinary stone formation. For instance, individuals with urinary stones often experience concurrent UTI, regardless of stone type [5].

UTI and urinary stones are interrelated conditions, as stones may predispose to infection, while urease-producing bacteria can alter urine chemistry and directly induce struvite stone formation [6]. Patients with urinary stones have a higher risk of experiencing tract blockage, dilatation, and effusion, conditions that create a favorable environment for pathogen proliferation and may contribute to the development of UTI [7]. The production of biofilms on pre-existing stones, particularly when the urothelial barrier is compromised, is another theory about the pathogenesis of UTI in the context of urolithiasis [8]. Researchers have discovered that certain non-struvite (CaOx and CaPhos) stones have more bacterial populations than the surrounding urine, since the composition and surface of urinary stones create an optimal environment for bacterial adhesion and proliferation, ultimately leading to the development of chronic bacteriuria [9].

The development of infected stones is largely driven by urease-producing and other bacterial strains, which facilitate recurrent infections [3]. In particular, these bacteria accelerate the conversion of urea into ammonia, which subsequently contributes to an alkaline environment that promotes kidney stone formation and increases the risk of concurrent infections [8]. The complex relationship between urinary infections and stones is further exacerbated by the rise of antibiotic resistance, especially in bacteria exhibiting urease activity that are frequently linked to UTI [3,10]. A considerable number of urease-producing bacterial strains are pathogenic and linked to UTI, carrying a wide range of antibiotic resistance and virulence genes [11]. Microorganisms such as *Proteus*, *Providencia*, *Serratia*, *Morganella*, *Klebsiella*, *Pseudomonas*, and *Staphylococcus* spp. harbor a range of antibiotic resistance and virulence determinants, presenting substantial challenges for therapeutic intervention [3,10,12].

USD and UTI are potential risk factors for each other since bacterial colonization along with elevated urine pH contributes to both the formation and progression of new and existing stones [8]. Although numerous studies have extensively investigated risk factors for stone formation, stone composition, and the microbiological characteristics of urinary calculi, comparatively limited evidence is available regarding determinants of UTI development among patients with urolithiasis. Urolithiasis patients who have UTI are at a higher risk of severe complications after surgical procedures, including postoperative infections, systemic inflammation, and even death from septic shock [13]. This complex scenario emphasizes the intricate relationship between UTI and USD, highlighting the necessity for comprehensive approaches to explore the mechanisms underlying bacterial virulence and antimicrobial resistance in the setting of UTI and urolithiasis, with considerable implications for treatment approaches [3,14]. Current evidence on urine microbiological profiles and antimicrobial resistance patterns among younger versus older patients with urolithiasis remains limited and fragmented [1]. Clinically applicable risk factors and validated threshold values that could reliably stratify infection risk in stone formers remain insufficiently defined in the current literature. The primary aim of this study was to evaluate the prevalence and microbiological characteristics of UTI among patients with urolithiasis, by comparing urolithiasis patients with and without UTI. By evaluating clinical presentations, associated factors, and microbial profiles, we aim to improve our comprehension of this intricate relationship and help develop enhanced diagnostic and treatment approaches. In addition, comparisons with the non-urolithiasis group were performed to provide contextual background rather than to establish causal associations.

## 2. Materials and Methods

### 2.1. Study Design and Patient Selection

This retrospective observational study was conducted at Gaziantep University Hospital, a tertiary referral center located in southeastern Türkiye, and covered the period from January 2014 to December 2024. All urine cultures obtained from patients evaluated in the Urology Clinic during the study period were screened. A total of 23,241 urine culture results during the 11-year period were evaluated. Adult patients (≥18 years) with at least one urine culture and an assigned urological diagnosis were eligible for inclusion. Patients with missing data were excluded (<1%: unlikely to introduce meaningful selection bias). Urolithiasis was designated as the presence of stones within any segment of the urinary tract. Patients were categorized into two groups, those with and without urolithiasis, based on relevant International Classification of Diseases (ICD) codes; however, all ICD-based classifications were subsequently reviewed and verified by a urology specialist using available radiological imaging, clinical documentation, and laboratory findings to confirm active or clinically relevant urolithiasis. In the urolithiasis group, detailed clinical and laboratory parameters were collected, including demographic factors, causative microorganisms, urine pH, presence of urinary anomaly, presence of hydronephrosis, stone type, and ureteral double J stent presence and indwelling duration. In addition, the study was designed to assess predefined associations between demographic characteristics, clinical findings and laboratory parameters. All stones were categorized as either struvite or non-struvite based on Fourier-transform infrared spectroscopy results. Non-struvite stones included calcium oxalate, calcium phosphate, and uric acid stones and were grouped together for analytical purposes. In cases where stone material was not retrieved or analysis was not available, stone composition data were recorded as ‘No result’ and excluded from composition-based subgroup analyses. For the non-urolithiasis group, these parameters were not included in the primary analysis. The large cohort provides substantial statistical power and supports the generalizability of the findings even without detailed clinical stratification. Comprehensive phenotyping of this group was beyond the scope of the study.

### 2.2. UTI Evaluation

Within the two groups, those with and without urolithiasis, we identified patients who met the criteria for UTI. Colony count thresholds were interpreted according to clinical context, in line with guideline-based recommendations [15]. In non-catheterized patients, a urine culture yielding ≥ 10^5^ CFU/mL of a recognized uropathogen was considered significant when accompanied by supportive indicators of infection, including elevated leukocyte count in urine microscopy, compatible clinical findings, and the requirement for antibiotic therapy. Cases consistent with asymptomatic bacteriuria were excluded from the analysis. In certain conditions, e.g., catheterized patients, lower colony count thresholds, as low as ≥10^3^ CFU/mL, were considered clinically significant when supported by compatible clinical and laboratory findings. For each patient, only one urine culture was included, prioritizing the most predominant or clinically significant pathogen, while flora contaminants were regarded as non-growth. To evaluate the association between urolithiasis and UTI, an additional criterion was applied: urine cultures collected within 90 days of the urolithiasis diagnosis or patient admission were selected for analysis, as, 90-day intervals are frequently used in infection surveillance to define temporally related infection events [16].

### 2.3. Statistical Analysis

Statistical analyses were performed using SPSS version 29.0 (IBM Corp., Armonk, NY, USA). Continuous variables are presented as mean ± standard deviation, and categorical variables as frequencies and percentages. Comparisons between two groups were conducted using the independent samples *t*-test, and one-way ANOVA was used for three or more groups. Categorical differences were assessed with the chi-square test, with significant results further explored through percentage distributions.

Factors affecting UTIs were evaluated using univariate and multivariate logistic regression. Univariate logistic regression analyses were initially performed to identify variables associated with urinary tract infection. Variables with a *p* value < 0.05 in univariate analysis were subsequently included in the multivariate logistic regression model to identify independent predictors while controlling for potential confounding factors. The final multivariate logistic regression model was constructed using a forced entry approach, in which all eligible variables were entered into the model simultaneously to estimate their independent effects. Results are presented as odds ratios (ORs) with 95% confidence intervals (CIs) and corresponding *p*-values, with statistical significance defined as *p* < 0.05.

Receiver operating characteristic (ROC) curve analysis was performed to assess the discriminatory performance of urine pH and stent indwelling time in predicting urinary tract infection. Optimal cut-off values were determined using the Youden index.

## 3. Results

### 3.1. Assessment of Urolithiasis

Overall, 23,241 urine cultures obtained from 12,708 unique patients presenting to the Urology Clinic in an 11-year period were screened. Among these, 2904 patients were diagnosed with urolithiasis and 9804 patients had no diagnosis of urolithiasis. UTI status, microbiological profiles, and clinical associations were analyzed at the patient level, while individual culture results were used to support patient classification. Statistically significant differences were detected between patients with and without urolithiasis regarding sex, age, and the presence of UTI (*p* < 0.001 for all), and the demographic comparisons of the patients are presented in Table 1.

### 3.2. Assessment of UTI

In the non-urolithiasis group, analysis of the association between UTI and demographic variables revealed that microbial growth was significantly related to both age and, more prominently, to sex (Table 2). There was a statistically significant difference across age groups (*p* = 0.011), and the prevalence of UTI was significantly elevated in females relative to males (*p* < 0.001).

In the urolithiasis group, in accordance with the predefined inclusion criteria, UTI analyses were based on urine cultures collected within 90 days of the urolithiasis diagnosis or patient admission. The microbial growth was likewise found to be significantly associated with both age and sex. A statistically significant difference was observed with respect to age (*p* = 0.008), in both the urolithiasis and non-urolithiasis groups, the prevalence of UTI was significantly higher among females compared to males (*p* < 0.001) (Table 2).

In the non-urolithiasis group, the potential differences in age and sex across various types of microorganisms were evaluated. Analysis of variance revealed a statistically significant difference among age groups (*p* < 0.001), and chi-square analysis also demonstrated a statistically significant difference with respect to sex. The frequency of fungal isolates was notably higher in males and in older patients (Table 3).

In the urolithiasis group, no statistically significant difference was observed with respect to age. However, Chi-square analysis indicated a significant sex-related difference (Table 3). In this group, Gram-negative bacteria were more frequently isolated in females, whereas, in contrast to the non-urolithiasis group, fungal isolates were more common in males.

In the non-urolithiasis group, the most frequently isolated microorganisms, as causative agents of UTI, were *Escherichia coli* (52.3%), *Klebsiella pneumoniae* (12.6%), coagulase-negative *Staphylococcus* (5.4%), *Candida* spp. (4.9%), *Enterococcus faecalis* (3.9%), *Pseudomonas aeruginosa* (3.8%), and other bacteria (17.1%). In the urolithiasis group, the most common isolates were *E. coli* (52.3%), *K. pneumoniae* (10.4%), *E. faecalis* (6.6%), *P. aeruginosa* (5.8%), *Enterococcus faecium* (4.4%), *Candida* spp. (4.3%), *Proteus mirabilis* (3.6%), and other bacteria (12.7%). Statistically significant increases were observed in the prevalence of *P. aeruginosa* (*p* = 0.001), *E. faecalis* (*p* = 0.002), and *K. pneumoniae* (*p* = 0.002) in patients with stones. Additionally, *E. coli* was significantly more frequent in women, while *P. aeruginosa* was more frequent in men, in both groups.

### 3.3. Association Between UTI and Urolithiasis

In patients with urolithiasis, clinical and laboratory parameters were compared according to the presence or absence of UTI. The presence of a ureteral double J stent was strongly associated with infection, and stent indwelling duration was significantly longer in infected patients (30.10 ± 31.25 days) compared to non-infected patients (17.78 ± 13.95 days, *p* < 0.001), and urine pH was significantly higher in the infection group (6.37 ± 0.66 vs. 5.78 ± 0.42, *p* < 0.001). Hydronephrosis and urinary anomalies were also more common among patients with infection (*p* < 0.001 for both) (Table 4).

Univariate and multivariate logistic regression analyses were performed to evaluate the contribution of various clinical parameters to the risk of urinary tract infection (UTI) in patients with urolithiasis (Table 5). In univariate analysis, urine pH, the presence of hydronephrosis, Double J stent presence, stent indwelling time, urinary anomaly, and struvite stone composition were significantly associated with UTI. However, in the multivariate model, only urine pH, the presence of hydronephrosis, and Double J stent presence remained independently associated with UTI. In the multivariate analysis, urine pH was identified as an independent predictor of UTI (OR: 6.37, 95% CI: 2.67–15.19, *p* < 0.001), indicating a substantially increased risk of infection with higher urine pH levels. Similarly, the presence of hydronephrosis was independently associated with an increased risk of UTI (OR: 9.14, 95% CI: 3.74–22.35, *p* < 0.001). Although Double J stent presence was associated with an increased risk of UTI in univariate analysis (OR: 1.29, 95% CI: 1.09–1.55, *p* = 0.001), it demonstrated a protective association in the multivariate model (OR: 0.44, 95% CI: 0.19–0.99, *p* = 0.048) after adjustment for other covariates. This finding suggests that the apparent risk observed in the univariate analysis may be influenced by confounding clinical factors. Stent indwelling time was significantly associated with UTI in univariate analysis (OR: 1.04, 95% CI: 1.03–1.05, *p* < 0.001); however, this variable was not included in the multivariate model. Urinary anomaly and struvite stone composition were also significantly associated with UTI in univariate analyses; however, these associations did not remain statistically significant after multivariate adjustment.

**Figure 1:**
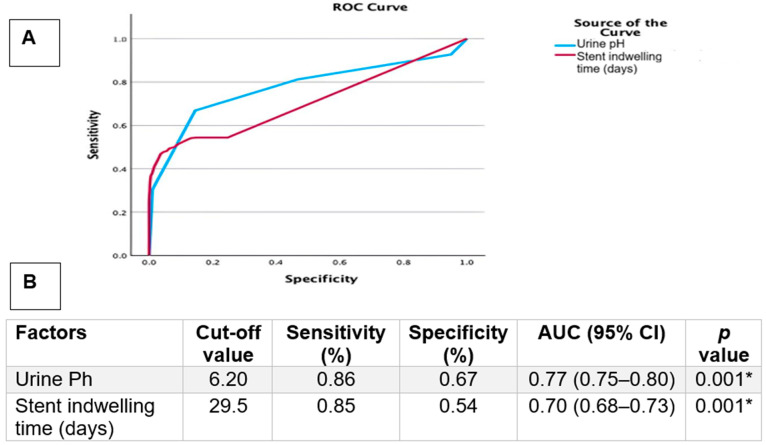
Receiver Operating Characteristic (ROC): (**A**) ROC curves illustrate the discriminative performance of urine pH and stent indwelling duration for UTI presence in patients with urolithiasis; (**B**) Parameters included in the ROC curve analysis. *: *p* < 0.05.

Post hoc comparisons demonstrated that patients with Gram-positive infections had significantly higher urine pH values compared to the Gram-negative and fungal groups. This finding was maintained in the multivariate model, indicating an association between urine pH and Gram-positive microorganisms. Receiver operating characteristic (ROC) curve analysis was performed to evaluate the discriminatory performance of urine pH and stent indwelling time for the presence of urinary tract infection. ROC analysis identified a urine pH cut-off value of 6.20, which yielded a sensitivity of 86% and a specificity of 67% (AUC: 0.77, 95% CI: 0.73–0.81, *p* = 0.001) (Figure 1). Similarly, a stent indwelling time cut-off value of 29.5 days demonstrated a sensitivity of 85% and a specificity of 54% (AUC: 0.70, 95% CI: 0.68–0.73, *p* = 0.001) (Figure 1). Given the relatively modest specificity values, particularly for stent indwelling time, these cut-off points should be interpreted as exploratory findings that may assist in risk stratification rather than definitive clinical thresholds. Struvite stones were observed more frequently in patients with urinary tract infection; however, this finding should be interpreted with caution due to incomplete stone composition data.

The ROC-derived cut-off values of urine pH and stent indwelling time were not internally or externally validated, which limits their immediate clinical applicability and supports their interpretation as exploratory findings.

## 4. Discussion

Urolithiasis represents a significant global health concern, with reported prevalence rates reaching up to 14.8% and recurrence rates approaching 50% within five years of the first occurrence [17,18]. Beyond its direct clinical burden, stone disease is increasingly acknowledged as a multifactorial condition in which systemic comorbidities, metabolic disturbances, and microbiological factors interact in complex ways [19,20]. Our findings confirm well-established demographic patterns, including sex and age-related variances, while highlighting novel associations between urolithiasis, UTI, invasive procedures, and systemic conditions. The interplay between UTI and urolithiasis has long been debated. UTI contributes to stone disease through multiple mechanisms. Bacteria exhibiting urease activity such as *Proteus*, *Klebsiella,* and *Pseudomonas* hydrolyze urea into ammonium, generating alkaline urine that favors crystallization of struvite and apatite [21]. Within alkaline urine (pH > 7.2), magnesium ammonium phosphate readily crystallizes, driving rapid stone growth in the presence of obstruction or foreign bodies [21]. Our findings revealed a particularly noteworthy observation: while a urinary pH of 7.2 was associated with stone formation, in individuals with pre-existing stones, a pH of 6.2 was significantly associated with UTI. Alkaline urine environments favor the growth of urease-producing and opportunistic pathogens, promote ammonium formation, and may impair host defense mechanisms within the urinary tract. In stone-forming patients, even modest alkalinization may reflect underlying obstruction, bacterial metabolic activity, or altered urinary flow, thereby facilitating infection. Interestingly, elevated urinary pH was more strongly associated with Gram-positive infections in urolithiasis patients.

While struvite stones are the classical hallmark of infection, accumulating evidence indicates that even non-struvite calculi may harbor bacteria. Urinary calculi often possess a rough, uneven surface that incorporates organic materials including proteins, glycoproteins, and cellular remnants, which together promote bacterial attachment and colonization [22]. This initial microbial colonization on the stone surface facilitates biofilm development and mineral entrapment, processes closely linked to persistent infections and recurrent stone formation [23]. Several studies have recovered pathogens, including *E. coli*, from stones previously considered sterile [24,25]. Biofilm-forming pathogens anchor mineralized crystals within their extracellular matrix, accelerating lithiasis and sustainment [26]. Even non-urease producers, such as *E. coli*, can promote calcium oxalate aggregation through surface interactions and urinary citrate reduction [26]. Emerging data underscore the urinary microbiome as a pivotal contributor to the development of urinary stones. Antibiotic-induced dysbiosis can deplete protective taxa such as *Oxalobacter formigenes* and *Lactobacillus*, while favoring colonization by biofilm-forming bacteria or microorganisms with urease activity [26]. This underscores the dual role of antibiotics: while critical for infection control, they may inadvertently exacerbate long-term stone risk.

Urinary stones may increase the risk of UTI whether they themselves are infected or not. Chronic infection further amplifies this cycle through the induction of inflammatory mediators such as IL-1β, IL-6, and IL-8, which disrupt urinary homeostasis and facilitate crystal retention [27,28]. This supports the hypothesis that UTIs may contribute to ammonium-free stone formation [29]. Conversely, obstruction and stone-related stasis predispose to recurrent infections, sometimes culminating in life-threatening urosepsis if not promptly recognized and treated [21]. Also, some data suggest that infections may precede rather than follow stone development [24]. Therefore, patients with persistent UTIs should be evaluated for underlying calculi [30]. As a limitation, although stone composition analysis was not available for all patients with urolithiasis in our study, it should be emphasized that regardless of whether the stones are struvite or non-struvite, all stone-bearing patients require close monitoring for UTI. However, stone-type–specific findings should be interpreted as hypothesis-generating rather than confirmatory.

Previous literature demonstrated strong associations between urolithiasis and chronic conditions such as diabetes, hypertension, and metabolic syndrome, with gender-specific patterns showing higher cardiovascular and metabolic comorbidities in males and increased diabetes and thyroid dysfunction in females [31,32,33]. Prior microbiome-based studies have also demonstrated higher bacterial abundance in female stone formers [34]. Consistent with our findings, in previous large-scale studies *E. coli* remained the most prevalent uropathogen, particularly among women [1]. In contrast, *E. faecalis* predominated in older men, likely reflecting age-related prostatic dysfunction and frequent catheterization [1,35]. Also in our study, statistically significant increases were observed in prevalence of *E. faecalis* (*p* = 0.002) in urolithiasis patients. These gender-based microbial differences suggest that empirical antibiotic therapy should consider patient sex to limit the risk of multidrug resistance [1]. In both the urolithiasis and non-urolithiasis groups, UTIs were more prevalent in females than males; however, UTI prevalence increased in the presence of urolithiasis for both sexes. Furthermore, elderly individuals are at increased risk for urolithiasis attributable to age-related deterioration in renal function, the effects of polypharmacy, inadequate hydration, and decreased mobility [36,37]. Moreover, aging is linked to decreased diversity of the urinary microbiome, a higher prevalence of asymptomatic bacteriuria, and increased susceptibility to antibiotic-driven dysbiosis, collectively contributing to an increased risk of stone formation [38,39]. In our study, statistically significant difference was observed with respect to age (*p* = 0.008) in development of UTI in urolithiasis patients. These findings reinforce the concept that gender-specific and age-specific metabolic pathways contribute to stone pathogenesis, underscoring the need for personalized preventive strategies.

An important risk factor for UTI development in stone formers is urinary catheterization, a common intervention across medical specialties [40]. Catheter-associated UTI (CAUTI) remains the most common hospital-acquired infection worldwide [41]. Long-term indwelling stents significantly increase the risk of colonization, obstruction, and recurrent stone formation, as biofilm formation on catheter surfaces and ureteral stents facilitates both bacterial persistence and encrustation [30]. In our study, the presence of a ureteral double J stent was strongly associated with infection, and stent indwelling duration was markedly longer in infected patients (30.10 ± 31.25 days) compared to non-infected patients (17.78 ± 13.95 days, *p* < 0.001). This finding aligns with experimental and clinical evidence demonstrating rapid biofilm formation on stent surfaces, progressive bacterial colonization, and increasing antimicrobial tolerance over time. Importantly, the threshold identified in our analysis provides a practical temporal benchmark that may support earlier stent exchange or removal in high-risk patients. Prior evidence showed that catheterization not only predisposes to UTI but also alters antimicrobial susceptibility, particularly among ESBL-producing Enterobacterales [42]. *P. aeruginosa*, although less common than *E. coli*, remains clinically relevant due to its intrinsic multidrug resistance [40]. These observations highlight the need to minimize stent dwell times and employ improved biomaterials to reduce infection risk. Although ureteral double J stent presence was associated with lower odds of UTI (OR 0.44), this counterintuitive finding should not be interpreted as protective and likely reflects residual confounding, closer surveillance, earlier antimicrobial treatment, or overadjustment.

Hydronephrosis is a significant risk factor for infection in patients with urolithiasis [43]. When hydronephrosis is accompanied by fever, it raises the suspicion of a UTI, typically necessitating hospitalization and intravenous antibiotic therapy, and in some cases, urgent surgical intervention may be required [6]. In our study, urine pH and the presence of hydronephrosis were independently associated with microbial growth in urolithiasis patients. Interestingly, hydronephrosis was observed in 32.4% of Gram-positive (n = 68), 37.6% of Gram-negative (n = 282), and 72.1% of fungal infections (n = 31), with a statistically significant higher rate in fungal cases (*p* = 0.001). Another notable finding is that urinary anomalies were associated with Gram-negative microbial growth (*p* = 0.008).

Urinary stones themselves may present with persistent or recurrent infections, as bacteria colonize the interstices of calculi. Manipulation during lithotripsy or the presence of obstruction may lead to bacteremia, urosepsis, and occasionally death, despite careful technique and prophylactic antibiotic administration [30]. UTI in the setting of urolithiasis should be considered high-risk events requiring prompt antimicrobial therapy, even before culture results are available [43]. Stone removal remains a cornerstone in the management of recurrent infections, since infection often persists until the nidus is eliminated [30]. Empirical antibiotics should be tailored to patient sex and catheter status, considering documented differences in microbial spectrum and resistance [1]. Finally, preventive strategies must integrate metabolic, microbiological, and procedural risk factors, recognizing urolithiasis as a disorder of systemic and ecological imbalance rather than a purely mechanical problem. A major strength of our study is the comprehensive evaluation of demographic, metabolic, microbiological, and procedural factors in a large patient cohort. However, several limitations warrant mention. This was a retrospective, single center study conducted at a tertiary referral hospital in Türkiye, which may introduce both selection and information bias and limits the generalizability of our findings to populations with distinct genetic profiles, healthcare settings, and environmental contexts. Also, a key limitation of this study is the limited clinical phenotyping of the non-urolithiasis cohort. Therefore, comparisons involving the non-urolithiasis group should be interpreted as descriptive findings rather than causal associations. Another important limitation of this study is the inability to differentiate between upper and lower UTI or to stratify cases according to infection severity. The retrospective design also precludes causal inference regarding the interplay between comorbidities, infections, and stone composition. The lack of stone composition analysis for most patients limits definitive conclusions regarding struvite versus non-struvite stones.

## 5. Conclusions

In this large retrospective cohort, urolithiasis was associated with a significantly higher prevalence of UTI and distinct demographic and microbiological patterns. While UTIs were more frequent in females among patients with and without urinary stones, UTI prevalence increased in the urolithiasis group for both sexes. Gram-negative bacteria predominated overall; however, *P. aeruginosa*, *E. faecalis*, and *K. pneumoniae* were significantly more prevalent in patients with urolithiasis. Among patients with urolithiasis, UTI was strongly associated with ureteral double J stent presence, prolonged stent indwelling time, elevated urine pH, hydronephrosis, and urinary tract anomalies, with urine pH and hydronephrosis revealing an independent association. These findings support the concept that urolithiasis is not merely a local urinary pathology but a condition shaped by host, microbial, and procedural factors. From a clinical standpoint, the factors identified in this study may support a more individualized approach to infection risk assessment in patients with urolithiasis. Recognition of these risk profiles may facilitate targeted prevention, timely diagnosis, and optimized management strategies to reduce infection-related morbidity in stone forming patients.

## Figures and Tables

**Table 1:** Distribution of demographic characteristics and UTI prevalence in patients with and without urolithiasis.

Parameter	Non-Urolithiasis (n: 9804)	Urolithiasis (n: 2904)	*p* Value
$\mathrm{Age}\;(\bar{x}$ ± sd)	43.10 ± 23.50	37.24 ± 21.32	^x^ <0.001 *
Sex (n, %)			
Male	5238 (53.4%)	1447 (49.8%)	^y^ <0.001 *
Female	4566 (46.6%)	1457 (50.2%)	
Urinary infection (n, %)			
Absent	6974(71.1%)	1901 (65.5%)	^y^ <0.001 *
Present	2830 (28.9%)	1003 (34.5%)	
Microorganism (n, %)			
Gram-positive	540 (19.1%)	210 (20.9%)	^y^ 0.347
Gram-negative	2150 (76%)	750 (74.8%)	
Fungus	140 (4.9%)	43 (4.3%)	

^x^: Independent Samples *t*-test; ^y^: Chi-Square Analysis; *p* < 0.05 *; Microorganism distribution refers only to patients with positive urine cultures.

**Table 2:** Differences in urinary infection rates according to the demographic structure of urolithiasis and non-urolithiasis patients.

	Non-Urolithiasis			Urolithiasis		
Parameter	No UTI (n: 6974)	UTI (n: 2830)	*p* Value	No UTI (n: 1901)	UTI (n: 1003)	*p* Value
$\mathrm{Age}\;(\bar{x}$ ± sd)	42.75 ± 22.63	43.95 ± 25.52	^x^ 0.011 *	37.94 ± 20.53	35.92 ± 22.70	^x^ 0.008 *
Sex (n, %)				1082 (74.8%)		
Male	3948 (75.4%)	1290 (24.6%)	^y^ <0.001 *		365 (25.2%)	^y^ 0.001 *
Female	3026 (66.3%)	1540 (33.7%)		819 (56.2%)	638 (43.8%)	

^x^: Independent Samples *t*-test; ^y^: Chi-Square Analysis; *: *p* < 0.05; *p* values indicate within-group comparisons.

**Table 3:** Distribution of UTI causative microorganisms according to age and sex in patients with and without urolithiasis.

	Non-Urolithiasis				Urolithiasis			
Parameter	Gram-pos. (n: 540)	Gram-neg. (n: 2150)	Fungus (n: 140)		Gram-pos. (n: 210)	Gram-neg. (n: 750)	Fungus (n: 43)	
$\mathrm{Age}\;(\bar{x}$ ± sd)	40.4 ± 25.0	44.4 ± 25.6	50.7 ± 23.8	^x^ 0.001 *	35.0 ± 22.5	35.8 ± 22.8	41.3 ± 20.5	^x^ 0.250
Sex (n, %)								
Male	237 (18.4%)	971 (75.3%)	82 (6.4%)	^y^ 0.001 *	87 (23.8%)	254 (69.6%)	24 (6.6%)	^y^ 0.003 *
Female	303 (19.7%)	1179 (76.5%)	58 (3.8%)		123 (19.3%)	496 (77.7%)	19 (3.0%)	

Gram-pos.: Gram-positive; Gram-neg.: Gram-negative; ^x^: Anova Analysis; ^y^: Chi-Square Analysis; *p* < 0.05 *. Percentages are calculated within sex category.

**Table 4:** Comparison of clinical and laboratory parameters based on microbial growth in patients with urolithiasis.

	Urolithiasis		
Parameter	No UTI (n: 1901)	UTI (n: 1003)	*p* Value
$\mathrm{Age}\;(\bar{x}$ ± sd)	37.94 ± 20.53	35.92 ± 22.70	^x^ 0.008 *
Sex (n, %)			
Male	1082 (56.9%)	365 (36.4%)	^y^ 0.001 *
Female	819 (43.1%)	638 (63.6%)	
Hydronephrosis			
Absent	1695 (73.2%)	622 (26.8%)	^y^ 0.001 *
Present	206 (35.1%)	381 (64.9%)	
Double J Stent			
Absent	1431 (75.8%)	457 (24.2%)	^y^ 0.001 *
Present	470 (46.3%)	546 (53.7%)	
Stent indwelling time (days)	17.78 ± 13.95	30.10 ± 31.25	^x^ 0.001 *
Urinary Anomaly			
Absent	1758 (66.7%)	879 (33.3%)	^y^ 0.001 *
Present	143 (53.6%)	124 (46.4%)	
$\mathrm{Urine}\;\mathrm{pH}\;(\bar{x}$ ± sd)	5.78 ± 0.42	6.37 ± 0.66	^x^ 0.001 *
Stone analysis			
Non-struvite Stone	95 (64.6%)	52 (35.4%)	^y^ 0.001 *
Struvite	7 (18.9%)	30 (81.1%)	
No Result	1799 (66.1%)	921 (33.9%)	

^x^: Independent Samples *t*-test; ^y^: Chi-Square Analysis; *: *p* < 0.05; Stone composition data were unavailable for a substantial proportion of patients.

**Table 5:** Univariate and multivariate analysis of factors associated with urinary infection.

	Urinary Infection			
	Univariate		Multivariate	
Variable	OR (95% CI)	*p* Value	OR (95% CI)	*p* Value
Urine pH	2.04 (1.53–2.72)	0.001 *	6.37(2.67–15.19)	0.001 *
Hydronephrosis				
Absent	1	-	1	-
Present	1.61 (1.15–2.25)	0.001 *	9.14 (3.74–22.35)	0.001 *
Double J Stent				
Absent	1	-	1	-
Present	1.29 (1.09–1.55)	0.001*	0.44 (0.19–0.99)	0.048 *
Stent indwelling time (days)	1.04 (1.03–1.05)	0.001 *		
Urinary Anomaly				
Absent	1	-	1	-
Present	1.55 (1.23–2.64)	0.008 *	1.53 (0.55–4.23)	0.408
Stone analysis				
Struvite	2.05 (1.21–3.19)	0.001 *	1.46 (0.40–5.28)	0.559

OR: odds ratios; CI: confidence intervals; *: *p* < 0.05. Variables with *p* < 0.05 in univariate analysis were included in the multivariate logistic regression model. Stent indwelling time was not included in the multivariate model to avoid redundancy with Double J stent presence.

## Data Availability

The data that support the findings of this study are available from the corresponding author upon reasonable request.

## Abbreviations

The following abbreviations are used in this manuscript:

USD	Urinary stone disease
UTI	Urinary tract infection
ICD	International Classification of Diseases
OR	Odds ratios
